# Metabolic Features of Multiple Myeloma

**DOI:** 10.3390/ijms19041200

**Published:** 2018-04-14

**Authors:** Chaima El Arfani, Kim De Veirman, Ken Maes, Elke De Bruyne, Eline Menu

**Affiliations:** Department of Hematology and Immunology, Myeloma Center Brussels, Vrije Universiteit Brussel (VUB), 1090 Brussels, Belgium; Chaima.El.Arfani@vub.be (C.E.A.); kdeveirm@vub.ac.be (K.D.V.); ken.maes@vub.be (K.M.); Elke.De.Bruyne@vub.be (E.D.B.)

**Keywords:** metabolism, multiple myeloma, bone marrow micro-environment

## Abstract

Cancer is known for its cellular changes contributing to tumour growth and cell proliferation. As part of these changes, metabolic rearrangements are identified in several cancers, including multiple myeloma (MM), which is a condition whereby malignant plasma cells accumulate in the bone marrow (BM). These metabolic changes consist of generation, inhibition and accumulation of metabolites and metabolic shifts in MM cells. Changes in the BM micro-environment could be the reason for such adjustments. Enhancement of glycolysis and glutaminolysis is found in MM cells compared to healthy cells. Metabolites and enzymes can be upregulated or downregulated and play a crucial role in drug resistance. Therefore, this review will focus on changes in glucose and glutamine metabolism linked with the emergence of drug resistance. Moreover, metabolites do not only affect other metabolic components to benefit cancer development; they also interfere with transcription factors involved in proliferation and apoptotic regulation.

## 1. Introduction

In this review, we focus on the mechanisms of cancer metabolism in multiple myeloma (MM), which is a hematologic malignancy described as an expansion of dysfunctional plasma cells in the bone marrow (BM). This expansion and accumulation of malignant plasma cells is linked with high levels of monoclonal proteins (M-spike) in serum and urine [1]. The disease is usually preceded by a premalignant condition known as monoclonal gammopathy of undetermined significance (MGUS), whence MM arises [1,2]. The expansion of malignant plasma cells and the production of M-protein in excess causes the following frequently observed symptoms in MM: hypercalcemia, renal failure, anaemia and bone lesions (CRAB features) [3,4]. Reports estimate yearly 41,719 new cases in Europe and 20,462 deaths. Worldwide, MM affects 1–5 per 100,000 individuals every year. Despite the progress in treatment, MM remains an incurable disease and the prevalence continues to increase due to the ageing population, with a median age of 73–75 at diagnosis [5,6,7]. Early therapies were the combination of melphalan with prednisone followed by melphalan and autologous hematopoietic stem cell transplantation. Over the last decade, novel therapies improved survival in MM patients, such as the proteasome inhibitor bortezomib and the immunomodulatory drugs (IMiDs) thalidomide, lenalidomide and pomalidomide. Also, promising preliminary results with the monoclonal antibody daratumumab have been shown. These drugs are also combined with dexamethasone, an immunosuppressive drug [8,9]. The rate of complete response and the overall survival have improved with these drugs [9]. Despite these improvements in overall survival, MM patients eventually relapse. The main cause of this relapse is the development of drug resistance. This can be either intrinsically by mutational changes in genes or ribosomal proteins (monoallelic loss of 60S ribosomal proteins) where decreased ribosomal proteins are correlated with tumour progression and drug resistance; or extrinsically by the BM micro-environment [10,11].

It is well known that tumour growth and metastasis are increased by intense and complex neovascularisation in solid tumours [12,13]. Similarly, despite MM being a non-solid tumour situated in the BM, blood vessel development is increased through secretion of cytokines such as vascular endothelial growth factor (VEGF) and fibroblast growth factor-2 (FGF-2). Also, interleukin-6 (IL-6) is secreted by BM endothelial cells, which promote cell growth in MM cells. This causes an increase in vascularisation, which promotes MM progression and is correlated with poor survival. While anti-angiogenic therapy has been investigated as anti-cancer agent in MM, this could have some side effects. Angiogenesis leads to higher oxygen access, resulting in a less hypoxic environment. The use of anti-angiogenic therapy would therefore result in a more hypoxic environment, together with higher glycolysis rates of the tumour cells [1,14,15,16].

Besides the effect of IL-6, there is a synergistic activity of IL-6 and IL-3, leading to plasma cell differentiation from peripheral blood mononuclear cells [17].

The BM micro-environment consists of two different compartments: a cellular and a non-cellular compartment. The former consists of hematopoietic cells important for the immune system on the one hand and non-hematopoietic cells such as bone marrow stromal cells (BMSC), fibroblasts, osteoblasts and blood vessels on the other hand [1,18]. The non-cellular compartment consists of fibronectin, laminin and collagen [19]. Malignant cells have an impact on the functionality of the BM micro-environment that favours their survival and growth. Indeed, MM cells interact with BMSC by adherence, resulting in the activation of cell cycle pathways and anti-apoptotic pathways such as Janus kinase (JAK)/signal transducer and activator of transcription 3 (STAT3), which leads to upregulation of the anti-apoptotic proteins BcL-xL and Mcl-1. NF-κB signaling is also activated with inhibitors of apoptosis proteins (IAP) as an anti-apoptotic protein [1,20]. These effects of MM cells on the BM can explain the presence of anaemia in MM patients since the infiltrating MM cells can be disadvantageous to marrow erythropoietic niches, which could lead to erythroid cell apoptosis due to MM cells producing specific cytokines, such as Fas ligand (FL), tumour necrosis factor (TNF) and TNF-related apoptosis-inducing ligand (TRAIL) [21].

MM is further characterised by osteolysis, whereby malignant plasma cells activate osteoclast (OC) progenitors and initiate osteoclastic bone resorption. Osteoclast formation is stimulated by the adherence of MM cells in the BM via vascular cell adhesion molecule 1 (VCAM-1) and α4β1 integrin. Together with the receptor activator of nuclear factor κB ligand (RANKL), they belong to the osteoclastogenic factors inducing osteolysis [22,23]. Furthermore, interleukin-1β (IL-1β) and TNF-β, secreted by the malignant plasma cells, are defined as osteoclast-activating factors (OAF) due to their osteoclast activation and bone reabsorption function [24].

Besides the changes induced by MM cells in the BM micro-environment, these malignant plasma cells experience metabolic changes themselves compared to healthy plasma cells. Known metabolic rearrangements in MM cells are adjustments in the glucose pathway, glutamine pathway, serine metabolism, pentose phosphate pathway (PPP) and folate pathway. Furthermore, the overall changes in cell metabolism and the BM environment induce drug resistance in MM.

## 2. General Cancer Metabolism

Every healthy cell is obliged to import nutrients from the environment to fulfil biosynthetic demands. This allows proliferation, differentiation and migration. Glucose and glutamine are indispensable for survival of mammalian cells [25]. Indeed, glucose can enter the cell through different glucose transporters: GLUT1 (erythrocytes, vascular endothelium), GLUT2 (hepatocytes, pancreatic β-cells, intestinal mucosa and renal cells), GLUT3 (neurons) and GLUT4 (skeletal and cardiac muscle), wherein each membrane protein is expressed depending on the cell type [26]. The process of glycolysis starts once glucose enters the cell; it goes through several conversions in the cytosol, ending in the generation of 2 moles pyruvate and 2 moles adenosine triphosphate (ATP). At first glucose is converted into glucose-6-phosphate after addition of a phosphate group, which prevents the efflux of glucose. Fructose-6-phosphate is formed and followed by fructose-2,6-biphosphate, d-glyceraldehyde 3-phosphate, 1,3-biphosphoglycerate, 3-phosphoglycerate, 2-phosphoglycerate and phosphoenolpyruvate (PEP). In a final step, pyruvate is formed after the conversion of PEP, which is catalysed by pyruvate kinase (PK) [27].

**Figure 1:**
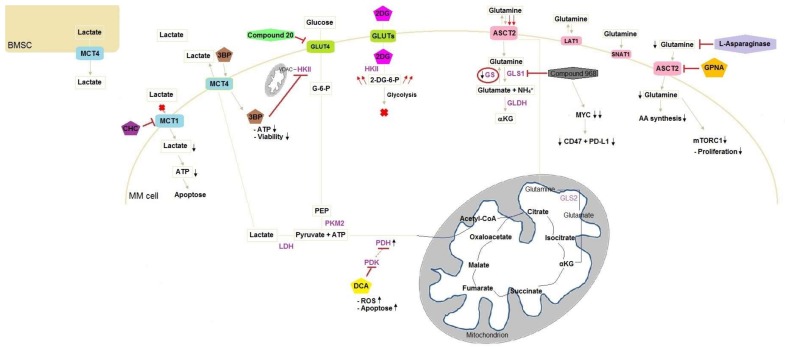
Schematic presentation of targets in glucose and glutamine metabolism in MM cells. Metabolic rearrangements in MM cells after administering compounds inducing stimulation or inhibition. 3BP enters the cell through MCT4 and inhibits HKII connected to the outer mitochondrial membrane via VDAC leading to ATP reduction and the loss of viable cells. 2DG enters the cell through GLUT and is phosphorylated by HKII. The phosphorylated form cannot be metabolised and accumulates by consequence leading to glycolysis blockage. Compound 20 inhibits GLUT4 resulting in chemosensitising. The inhibition of PDK on PDH is suppressed after addition of DCA to MM cells, which inhibits PDK. As a result, PDH activity increases and lactate production decreases, whereby TCA activity is increased. CHC is a MCT1 competitive inhibitor and blocks the entrance of lactate into the cell. This lack of fuel results in apoptosis due to ATP reduction. However, BMSC supply MM cells with lactate. Glutamine enters the cell through three transporters: ASCT2, LAT1 and SNAT1, with ASCT2 being the major glutamine transporter. Compound 968 inhibits GLS inducing MYC degradation and decrease of CD47 and PD-L1. MM cells lack GS, resulting in low intracellular glutamine concentrations and leading to higher glutamine influx. GPNA is a glutamine transporter inhibitor and induces a lack of glutamine intracellularly, which disadvantages AA synthesis and reduces mTORC1 activity, resulting in less cell proliferation. Instead of inhibiting glutamine transporters, l-asparaginase hydrolyses glutamine. AA, amino acid; BMSC, bone marrow stromal cells; 3BP, 3-bromopyruvate; CHC, α-cyano-4-hydroxycinnamic acid; DCA, dichloroacetate; 2DG, 2-deoxyglucose; 2-DG-6-P, 2-deoxyglucose-6-phosphate; G-6-P, glucose-6-phosphate; GLDH, glutamate dehydrogenase; GLS1, glutaminase 1; GLS2, glutaminase 2; GPNA, l-γ-glutamyl-p-nitroanilide; GS, glutamine synthetase; HKII, hexokinase II; LDH, lactate dehydrogenase; MM, multiple myeloma; MYC, myelocytomatosis oncogene cellular homolog; PDH, pyruvate dehydrogenase; PDK, pyruvate dehydrogenase kinase; PEP, phosphoenolpyruvate; PKM2, pyruvate kinase M2; ROS, reactive oxygen species; VDAC, voltage-dependent anion channel; 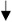, reduction; 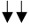, strong reduction; 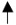, increase; 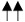, strong increase.

Next, pyruvate comes into the matrix of the mitochondria and is oxidised to acetyl-CoA, by the enzymatic activity of pyruvate dehydrogenase (PDH), which is the beginning of the tricarboxylic acid cycle (TCA), also called the Krebs cycle, under aerobic conditions. The fusion of acetyl-CoA with oxaloacetate forms citrate as the next metabolite in the TCA cycle [28]. Next, isocitrate is generated, followed by α-ketoglutarate (αKG), succinate, fumarate, malate and oxaloacetate (Figure 1). The cycle is completed by arrival at oxaloacetate and then starts over again [29]. Each rearrangement step releases energy, in the form of electrons, which are accepted by the so-called electron shuttles such as nicotinamide adenine dinucleotide (NAD^+^) and flavin adenine dinucleotide (FAD). These molecules are responsible for the transportation of the high-energetic electrons. When energy is released, the electron shuttles capture the energy and are reduced to nicotinamide adenine dinucleotide hydrogen (NADH) and FADH_2_. At the end, the electron shuttles are transported to the electron transport chain, located in the inner membrane of the mitochondria, to generate ATP [30]. The oxidative phosphorylation (OXPHOS) is a very efficient mechanism that generates 36 moles ATP out of 1 mole glucose in aerobic conditions. However, under anaerobic conditions, lactate is generated as a result of the reduction of pyruvate in the cytosol and then excreted out of the cell through monocarboxylate transporters (MCTs) [28]. The reduction of pyruvate into lactate is realised through lactate dehydrogenase (LDH).

Besides glucose, glutamine is by far one of the most abundant amino acids and crucial for the maintenance and promotion of normal cell function. Glutamine enhances proliferation, differentiation, cytokine production and apoptosis. Also, it is the precursor for nucleotide and nucleic acid synthesis [31]. First, glutamine can be transported into the cell via several transporters such as the neutral amino acid transporter (ASCT2) (Figure 1). After the passage into the cell, glutamine can then be used for biosynthesis [32,33]. The process of glutaminolysis takes place in two different parts of the cell similar to glucose metabolism: the cytosol and mitochondria. First, glutamine can be converted to glutamate by glutaminase 1 (GLS1) in the cytosol. In the mitochondria it is converted by glutaminase 2 (GLS2), where it can further be oxidised to αKG by glutamate dehydrogenase (GLDH) or aminotransferases and then participate in the TCA cycle as explained above by generating mitochondrial NADH, NADPH and ammonia [29,34,35,36]. Besides the participation in the TCA cycle, glutamine is also used in biosynthesis of nucleic acids [36]. Formation of purine nucleotides start with the conversion of 5-phospho-α-ribosyl-1-pyrophosphate (PRPP) by glutamine PRPP amidotransferase where glutamine donates an amino group to PRPP and attributes further a nitrogen and ends in inosine monophosphate, which is a fully formed purine nucleotide, after a series of reactions. In this nucleic acid synthesis, glycine, aspartate, ATP and tetrahydrofolate are also used [37,38].

The entrance of glucose, glutamine and various other nutrients into the cell is the very start of this complex metabolic machinery, crucial for the cellular respiration that sparks the conversion of these nutrients into biochemical energy. When comparing glycolysis with glutaminolysis, both processes result in energy production and nucleotide synthesis. The difference is that glycolysis provides more lactate and reduces mitochondrial atrophy. Glutaminolysis results in amino acid synthesis and fatty acid synthesis. These mechanisms together contribute to the growth and survival of the cancer cell [39]. However, cancer cells are characterised by an altered metabolism and this varies from one cancer type to another. Due to the high heterogeneity of the disease, the metabolic features are very diverse depending on the cancer type [28,40]. Nevertheless, most malignant cells have an enhanced aerobic glycolysis, also known as the ‘Warburg effect’, in common. This effect is described as the conversion of glucose to lactate in the presence of oxygen [41]. As tumour cells often reside in a hypoxic environment, the constitutive and high glycolytic flux is probably an adaptation of the malignant cells to the environmental stress [30]. The mitochondrial OXPHOS was considered as weakened or impaired by Warburg for the explanation of the high glycolytic flux of cancer cells. However, recent investigations have found that the mitochondrial OXPHOS mechanism remains unharmed and that OXPHOS is suppressed due to the enhanced glycolysis rather than a defect in the mechanism [28,42]. This effect is reversible: when glycolysis is inhibited, mitochondrial OXPHOS can regain its function, according to observations made by Fantin et al. [28,43]. Further, the increased glucose consumption can be used as a successful diagnostic tool via the imaging technique positron emission tomography (PET) where a glucose analogue, radioactively fluorine labelled (^18^F-fluorodeoxyglucose (^18^F-FDG)), is used as a tracer [25,41].

After glucose, glutamine is most often used for energy in cancer cells. One of the processes that provide anaplerotic flux is glutaminolysis, through the generation of αKG [29]. The amino acid is responsible for various important formations of components that are used for cell proliferation [44]. As described earlier in this review, glutamine enters the cell through many different transporters, and can directly be useful as such by its amido nitrogen present in the molecular structure of this amino acid for hexosamine and nucleotide synthesis in cancer cells [44,45]. In a further step, glutamine can be converted to glutamate and then to αKG that enters the TCA cycle for energy production. Furthermore, *de novo* synthesis of glutamine has been shown by He et al. in C6 glioma cells in which glutamine synthetase (GS), located in the cytoplasm, catalyses glutamine synthesis through ammonia and glutamate [44,46]. He et al., also reported that when C6 cells were deprived of glutamine, GS expression was upregulated and caused de novo glutamine synthesis [46]. Moreover, this observation can be useful in PET tracer studies: ^13^N-ammonia is taken up by tumour cells executing de novo glutamine synthesis and this gives information about the glutaminolysis rate in tumour cells [46]. Another hallmark is the capacity of glutamine to import essential amino acids. Indeed, Nicklin et al. showed that the entry of l-leucine (an essential amino acid) via the human l-type amino acid transporter 1 (LAT1) causes efflux of glutamine at the same time [47]. Glutamine can import other essential amino acids through that same mechanism [28].

Additionally, it is important to mention that the extensive glucose and glutamine uptake in cancer cells is a result of extracellular stimuli such as growth factor signaling [48]. A cell deprived of growth factors is shown to be negatively affected in terms of cell size and ATP generation despite the presence of glucose in medium. The cell is unable to maintain normal cellular bioenergetics, which can lead to activation of programmed cell death [49]. Overall, it is evident that environmental factors alter tumour metabolism in several cancers.

Besides the importance of glucose and glutamine metabolism in cancer, the folate metabolism is also linked with cancer. It is reported that low folate levels promote carcinogenesis and are associated with cytogenetic abnormalities. Moreover, low folate levels are shown to play a role in the neoplastic process [50]. Next to folate, proline also has importance in cancer. Proline is an amino acid with high abundance in the micro-environment. Proline dehydrogenase/oxidase (PRODH/POX) catalyses the conversion of proline into pyrroline-5-carboxylate (P5C). During this conversion, PRODH/POX donates an electron to the electron transport chain and results in the generation of reactive oxygen species (ROS). This initiates apoptosis and inhibition of tumour growth and cell proliferation, which can be useful as a target in cancers. However, proline biosynthesis, enhanced by myelocytomatosis oncogene cellular homolog (MYC) through glutamine, contributes to tumorigenesis. Indeed, MYC stimulates glutaminolysis through miR-23a/b, which is connected with proline synthesis [51].

## 3. Glucose Metabolism in multiple myeloma (MM)

In cancer research, glucose metabolism is the most studied branch in cancer metabolism. However, glycolysis has not yet been fully elucidated in MM. A first interesting enzyme in the glycolysis pathway is hexokinase II (HKII), which is part of four HKs isoforms. It is a widely overexpressed enzyme in several cancers including MM [52]. The hexokinase family irreversibly catalyses the first step of glycolysis, in which glucose is converted into glucose-6-phosphate after entering the cell through glucose transporters [36]. Investigations showed that HKII binds to the voltage-dependent anion channel (VDAC) present on the outer membrane of mitochondria [36,52] (Figure 1). This interaction is promoted by phosphoinositide-3 kinase (PI3K)/Akt signalling, resulting in stabilised high HKII levels leading to the continuous proliferation of malignant cells [52,53,54]. The constitutive overexpressed HKII can be inhibited by the small molecule 3-bromopyruvate (3BP) with alkylating properties. This compound was first identified as an inhibitor of glycolysis and oxidative phosphorylation [36,55] (Figure 1). The highly reactive molecule, which is a structural analogue of pyruvic acid, enters the cell through MCTs and releases a bromide radical after alkylation of the targeted protein [55]. As a result, lactate is not the only component that passes through MCTs. Reasons for the entry of 3BP are: (1) the abundant expression of MCTs; (2) possibly the similar molecular structure of 3BP and lactate; (3) high lactate efflux generating an acidic extracellular milieu, which benefits 3BP uptake in malignant cells [55,56].

Niedźwiecka et al. demonstrated morphological changes induced by the presence of 3BP in MM cells and flow cytometric analysis showed an increase in apoptotic MM cells after 2 and 4 h, both in a dose-dependent manner [57]. Also, ATP production and viability are reduced in MM cells after the addition of 3BP. Additionally, MM cell lines appear to be more susceptible to 3BP than leukaemic cell lines [42].

Interestingly, 2-deoxyglucose (2DG) displayed similar effects to 3BP on ATP production and cell survival in MM cells. This second anti-cancer agent is a glucose analogue and is phosphorylated by HKII into 2-DG-6-phosphate after entering the cell. The phosphorylated form cannot be metabolised and subsequently accumulates in the cell and interferes with the glycolytic pathway [58,59]. However, 2DG needs to be combined with other therapeutics due to its limited therapeutic effects as single agent [59]. Thereby, 3BP is shown to be a more convincing anti-cancer agent in terms of cell death and ATP depletion in MM cells than 2DG [52] (Figure 1).

After several enzymes and conversions, the final step of glycolysis consists of the conversion of phosphoenolpyruvate (PEP) into pyruvate and ATP, which is catalysed by PK in the cytosol of the cell. Similar to HKII, PK exists in four isoforms, where PKM2 seems to be upregulated in such a way that it becomes an abundant isoform in cancer cells [36,60]. PKM2 plays a supportive role in tumour progression and suppresses apoptosis [61]. Also, the *c-MYC* oncogene induces high PKM2 expression through never in mitosis (NIMA)-related kinase 2 (NEK2), which is a kinase that regulates chromosome segregation in the G2/M phase of the cell cycle [60]. Furthermore, the high enzymatic activity of PKM2 is associated with an increase of acetyl-CoA, whereas the opposite generates more lactate, leading to the Warburg effect [62]. It has recently been revealed that PKM2 expression is increased in MM cells. Moreover, silencing PKM2 leads to a decrease of MM cell growth and a cell cycle arrest at the G1/S transition [61].

Besides the formation of pyruvate, lactate is highly generated in cancer cells and transported out of the cell through MCTs. However, several papers have indicated that lactate can be incorporated into the cell and used as fuel for oxidative phosphorylation [63,64,65]. Influx and efflux depend on the concentration of lactate intra- and extracellularly and the presence of other substrates that bind to MCTs as well [65]. Myeloma cells express MCT1 to incorporate lactate in cytoplasm and generate ATP. Indeed, knockdown of the transporter leads to a decrease in lactate influx and lactate-derived ATP production, which induces apoptosis [66]. Besides knocking down MCT1, a competitive inhibitor (α-cyano-4-hydroxycinnamic acid (CHC)) of MCT1 is also capable of reducing the ability of MM cells to incorporate lactate into the cell in a dose-dependent manner [63]. This phenomenon can be accelerated through the addition of a pyruvate dehydrogenase kinase inhibitor (dichloroacetate (DCA)), which reaches its goal by shifting the glucose metabolism from pyruvate to acetyl-CoA instead of pyruvate to lactate [63,67,68,69,70] (Figure 1). Knowing that lactate is incorporated into MM cells suggests the presence of the latter in the micro-environment. Indeed, myeloma cells are supplied with lactate originating from the surrounding environment, which is described as the ‘reverse Warburg effect’ [63,66]. More precisely, BM-derived stromal cells secrete lactate through MCT4 in contrast to MCT1 [66,71,72]. While MCT1 inhibition leads to apoptosis due to a lack of fuel, MCT4 inhibition results in lactate accumulation, terminating in acidosis [73]. 

## 4. Glutamine Metabolism in MM

Glutamine is widely known as a non-essential amino acid playing a crucial role in different mechanisms in the human organism. As previously explained, glutamine enters the cell and is metabolised, resulting in different outcomes. It is converted into glutamate and ammonia (NH_4_^+^) through the activity of GLS1 and GLS2. In vitro, human myeloma cell lines (HMCLs) show an excess of NH_4_^+^ produced from glutamine, which leads to the assumption that MM cells are glutamine addicted [74]. Indeed, BM aspirates of MM patients have been checked for NH_4_^+^ levels in purified CD138^+^ cells. As predicted, there were significantly higher NH_4_^+^ levels in the presence of glutamine in CD138^+^ cells than in the CD138^−^ fraction [75]. It has also been shown that these malignant plasma cells lack GS and consequently rely on extracellular glutamine uptake, resulting in cytotoxic effects when glutamine is depleted. This lack of GS is further shown by the addition of methionine sulfoximine (MSO), a GS inhibitor, as the cytotoxic effects remained after glutamine depletion [74]. MM cells being highly dependent on glutamine could result in interesting therapeutic targets.

Next, l-asparaginase, a molecule used in the treatment of acute lymphoblastic leukaemia (ALL), has the capacity to hydrolyse glutamine, besides the degradation of asparagine, and leads to an intracellular depletion of amino acids and inhibition of mTOR activity [74,75,76]. Moreover, synergistic effects occur when l-asparaginase is combined with the proteasome inhibitor bortezomib, leading to increased cytotoxic effects in MM cells [74,77,78]. A second proteasome inhibitor, carfilzomib, also showed synergism with l-asparaginase, resulting in intensified anti-MM activity. IL-6 and insulin-like growth factor-1 (IGF-1), in combination with l-asparaginase and carfilzomib, did not reduce the anti-MM activity [76]. It is expected that the hypoxic environment of the BM, where the malignant plasma cells reside, should intensify the dependency on glutamine [77,78]. Affecting the glutamine transporter ASCT2 by inhibition with l-γ-glutamyl-p-nitroanilide (GPNA) and benzylserine decreases glutamine influx and results in lower proliferation rates [79] (Figure 1). Besides ASCT2, LAT1 and sodium-coupled neutral amino acid transporter 1 (SNAT1) are also major glutamine transporters expressed in MM cells. However, SNAT1 and LAT1 seem to contribute in a minor way to glutamine uptake [78]. Although optimal ASCT2 inhibitors are currently lacking, this target remains interesting due to the downstream effects, including the suppression of mTORC1 kinase activity, changes in cell proliferation, autophagy and protein synthesis [77,80].

The *MYC* oncogene is found in many human cancers and contributes to tumour growth, proliferation, DNA replication, transcription, protein biosynthesis and altered metabolism by escaping anti-tumoral mechanisms such as apoptosis, proliferative arrest and cellular senescence [81]. The transcriptional activity of MYC protein is upregulated in MM, more precisely during late stages of MM progression, and is correlated with poor survival [82]. Furthermore, MYC is involved in glutaminolysis, enhancing the expression of glutamine transporters and repressing inhibitors of glutaminolysis [82,83]. Also, glutamine consumption leads to accumulation of the oncometabolite 2-hydroxyglutarate via c-MYC [84]. It has been demonstrated that glutaminolysis inhibition results in apoptosis in HMCLs as well as the degradation of MYC. Indeed, inhibition of glutaminolysis with compound 968, which inhibits GLS, leads to MYC degradation. Also, glutamine removal from the media engenders degradation of MYC protein and apoptosis in HMCLs [82]. An interesting potential theory emerging from these findings is the possible susceptibility of MM cells to the immune response regulated by MYC. Degradation of MYC protein would enhance the antitumor immune response through reducing CD47 and PD-L1 [82,85].

Recent evidence has been shown that glutamine can influence proliferation, independent of glutamine metabolism. Depriving cancer cells from glutamine showed anti-proliferative effects without any rescue through addition of intermediate substrates generated in glutamine metabolism such as glutamate, 2-oxoglutarate and glutathione [33,86]. Moreover, Cacace et al., demonstrated that, while glutamine deprivation led to a reduction in glycolysis through downregulation of HIF-1α, activation of HIF-1α by dimethyl-2-oxoglutarate (DM-2-oxoglutarate) did not restore cell proliferation in cancer cells deprived of glutamine, indicating that the anti-proliferative effect occurs as a reaction to the absence of extracellular glutamine [86]. This leads to the conclusion that glutamine-induced cancer cell proliferation is not solely dependent on glutamine metabolism but also activation of other signalling pathways [86]. 

In this regard, Cacace et al., reported that glutamine activates (phosphorylates) the transcription factor STAT3 and controls hereby cell proliferation [86]. It seems that extracellular glutamine could activate certain cell surface receptors that can regulate STAT3. As a candidate, the authors suggest a receptor similar to Grp, a glutamine receptor identified in bacteria. This hypothesis is supported by the fact that other metabolites can also act on membrane receptors [86,87]. For example, lactate can activate GPR81, which is a G_i_-coupled receptor mainly expressed in adipocytes and has been shown to be highly expressed in different cancers. GPR81 silencing results in reduced tumour growth, decreased cell proliferation and lower mitochondrial activity when lactate is the only energy source [87]. This confirms the hypothesis on the actions of metabolites on membrane receptors. These findings were shown in breast cancer and cervical cancer; however, these investigations need to be refined and performed in MM [86,88].

## 5. Drug Resistance in MM

### 5.1. Standard-of-Care Drugs

In MM, altered tumour cell metabolism reduces the therapeutic effects of standard of care drugs such as bortezomib and melphalan due to drug resistance. The major reason for altered metabolism is the hypoxic tumour environment [89]. Indeed, HIF-1 is activated in a hypoxic environment and shifts glucose metabolism by intensifying the conversion of pyruvate into lactate instead of oxidation of pyruvate in the mitochondria. Mitochondrial energy production decreases as a result of HIF-1 activation [90]. It has been suggested that residual cells are resistant to treatment due to hypoxia in the BM, which leads to relapse. When comparing the level of HIF-1α and HIF-2α pathways by analysing gene-expression datasets between primary MM patients and healthy donors, a clear enrichment of HIF-1α and HIF-2α was observed in newly diagnosed MM patients [90,91,92]. Enrichment of these pathways was also observed in relapsed MM patients and bortezomib-refractory myeloma patients and was more abundant compared to bortezomib responding patients [90]. Additionally, HKII and lactate dehydrogenase A (LDHA) are found to be highly upregulated in relapsed MM patients compared to newly diagnosed myeloma patients, indicating increased glucose metabolism [90,93]. HMCLs exposed to hypoxic conditions and treatment with bortezomib, dexamethasone and melphalan showed an elevated glucose metabolism activity with overexpressed HIF-1α and LDHA after treatment [90]. The knowledge that enhanced glucose metabolism is linked with drug resistance, through HIF, generates interesting opportunities to inhibit glucose uptake in MM cells. Indeed, the combination of phloretin (GLUT1 inhibitor) and daunorubicin, a chemotherapeutic, enhanced the effect of the latter in hypoxia [90,94,95]. Wei et al. showed that compound 20 (GLUT4 inhibitor) results in chemosensitising to dexamethasone and melphalan of MM cell lines and patient material [96]. Besides GLUT1 as a target, targeting MM cells with ritonavir (GLUT4 inhibitor) increases the cytotoxic sensitivity and, together with the BH3 mimetic venetoclax, synergistic effects occur [97,98].

Next to targeting glucose uptake, HK also seems to be an interesting target. HK inhibitors such as 3BP, 2DG and lonidamine (LND) enhance drug response in normoxia in vitro; however, no response was observed in vivo [90,99,100]. Under hypoxic conditions, bortezomib decreased the activity of HKII whereas the activity of LDHA did not decrease, indicating a role for LDHA in bortezomib resistance. Moreover, bortezomib-resistant cells lose their resistance after LDHA knockdown leading to a reduction of lactate formation and should by consequence increase mitochondrial activity, reduce proliferation under hypoxic condition and thereby decrease tumorigenicity [43,90,101].

Bortezomib resistance does not only occur through LDHA and HIF. Actually, serine metabolism has recently been shown to have its own role in bortezomib resistance in MM. Serine biosynthesis starts either by extracellular import or via intracellular synthesis from glucose. The latter is the most used biosynthetic route to serine in many cancers. First, glucose enters the cell as previously explained and is metabolised via glycolysis. After several conversions, serine synthesis initiates from 3-phosphoglycerate (3-PG), which is converted into 3-phosphohydroxypyruvate (PHP) due to the enzymatic activity of phosphoglycerate dehydrogenase (PHGDH), the rate-limiting step in the serine synthesis pathway (SSP). Phosphoserine aminotransferase (PSAT) converts PHP into 3-phosphoserine (P-Ser) and is finally transformed into serine, catalysed by phosphoserine phosphatase (PSPH) [102]. SSP is shown to be beneficial to cancer cells due to its involvement in growth and proliferation [36,103]. PHGDH is upregulated in bortezomib-resistant HMCLs (shown in RPMI-8226) as well as PSAT and PSPH in different HMCLs. Similar to HIF, an overexpression of PHGDH and PSPH was observed in CD138^+^ cells aspirated from bortezomib-refractory myeloma patients compared to drug responsive MM patients. Depriving cells of serine was reported to be beneficial to bortezomib activity in RPMI-8226 cells [104]. Indeed, the absence of serine in the diet caused a reduction of tumour growth in mice [104,105]. These findings could result in an attractive way of tackling MM disease and can be used as a diagnostic tool to link overexpressed PHGDH with tumorigenesis.

Next to SSP, the PPP has been actively investigated in cancer. PPP consists of two phases: the oxidative and non-oxidative phase. The first phase starts from glucose-6-phosphate present in glycolysis metabolism and generates ribulose-5-phoshate after several conversions. Once ribulose-5-phoshate is formed, nucleotides are generated and pyrimidine and purine synthesis starts. The non-oxidative phase can be redirected into glycolysis metabolism by converting ribulose-5-phoshate into xylulose-5-phosphate and then into fructose-6-phosphate, which can further generate ATP through glycolysis [106,107]. Investigations demonstrated an upregulation of PPP in MM cells and together with the overexpression of SSP led to a higher antioxidant activity of bortezomib-resistant MM cells [104]. Chen et al. showed that PPP is upregulated when epidermal growth factor receptor (EGFR) inhibitors, such as gefitinib and afatinib, are administered leading to a limited response in MM cells [108]. As EGFR is found to be increased in some cancer types, targeting the latter can be a new attractive therapy when added to standard of care drugs [109]. However, the upregulation of PPP can be explained by the fact that EGFR inhibition triggers metabolic rearrangements as a compensatory mechanism to adapt and survive the loss of EGFR signalling. This resistance can be diminished through the addition of 6-aminonicotinamide (6-AN), an antimetabolite that inhibits NADPH supply in PPP combined with gefitinib [108,110].

Reasons for drug resistance can also be found in glutamine metabolism. As mentioned earlier, expression of GS is lacking in MM cells, whereas GLS expression is increased. Targeting GLS with the selective inhibitor CB-839 and combined with proteasome inhibitors (bortezomib, carfilzomib, ixazomib and oprozomib) enhanced the cytotoxic effects of proteasome inhibitors in vitro and in vivo, with carfilzomib showing the strongest synergy [111] (Table 1).

As mentioned before, the BM micro-environment and MM cells interact with one another and this interaction is known to be fundamental to the appearance of drug resistance in MM. Indeed, cell adhesion-mediated drug resistance (CAM-DR), a term introduced by Damiano and Dalton et al., is the manifestation of drug resistance after the adhesion to the extracellular matrix (ECM) [112,113,114]. This mechanism is diminished when PKM2 expression is promoted in MM cell lines and on the other hand it is supported after PKM2 knockdown [61]. PKM2 influences CAM-DR through the regulation of PI3/Akt and mitogen-activated protein kinase/extracellular signal-regulated kinase (MAPK/ERK1/2) signalling pathways, both known for their involvement in tumour progression [115,116,117]. Interestingly, the extracellular matrix glycoprotein Reelin leads to drug resistance by enhancing glycolysis and through HIF-1α [118].

### 5.2. Immunotherapy

Another form of therapy that is currently applied in MM is immune therapy. This consists of the use of the IMiDs lenalidomide and pomalidomide, immune checkpoint inhibitors, dendritic cells (DC)-based vaccines and allogeneic transplantation in MM patients [119]. The IMiDs can enhance the proliferation and function of NK and (NK) T cells. As mentioned earlier, the monoclonal antibody daratumumab also intensifies T cell immunity against myeloma [120]. Immune checkpoint inhibitors such as nivolumab, an anti-PD-1 antibody that targets the PD-1-PD-L1 pathway, enhance the antitumor T cell response. Furthermore, DC vaccines composed of DC fusion with tumour antigens are a potential mechanism to strengthen immunotherapy in MM [119]. Chimeric antigen receptor (CAR) T cell therapy is based on genetically modified autologous T cells, which express CAR and target specifically tumour antigens. Targets for CAR T cell therapy in MM have been developed and could be interesting for refractory MM patients [119,121].

Metabolic changes in the tumour micro-environment (TME) can also decrease the beneficial effects of immunotherapy [122]. The high rate of glucose and glutamine uptake, extensive lactate production and secretion shifts the TME into an advantageous place for tumour cells. However, this shaped TME is hostile to T cells due to nutrient deprivation, acidosis, accumulation of waste products and the hypoxic environment [122,123]. Acidification of the TME impairs T cell proliferation and decreases the NK cells’ functions. Buffering the pH improves immunotherapy outcomes with bicarbonate and can be useful in MM. Furthermore, anti-cancer agents such as 2DG are used in MM and shut glycolysis down; however, 2DG cannot be combined with immunotherapeutics due to the impairment of T cell metabolism and leads to decreased T cell antitumor effect. Similarly, DCA decreases the amount of lactate in the TME in MM, which could be a solution to the acidification issue. Unfortunately, DCA impairs the function of T cells [122]. Comparable to MM cells, immune cells utilise amino acids to be functional, such as l-arginine, which is a non-essential amino acid present in macrophages and DCs. When tumour cells secrete metabolites such as lactate, arginase is overexpressed and l-arginine is converted into urea and ornithine, leading to T cell dysfunction by interfering in the cell cycle progression. Since MM cells are known to secrete lactate, it is possible that MM cells could impair T cell function through this mechanism [124].

## 6. Conclusions and Future Perspectives

Metabolic changes are a general hallmark for most cancers. Investigating this field is a very complex task due to the large number of factors that have to be taken into account. However, an increasing number of papers on cancer metabolism have been published in the last decade. Cancer metabolism in MM is gradually being elucidated and new treatments have arisen from this process. The two most studied components in myeloma cells are glucose and glutamine, as in many other cancers. There are various targets present in glucose metabolism, as explained in this review. HKII is the first enzyme to catalyse the beginning of glucose metabolism. Together with other metabolites from glycolysis, these can be targeted with promising results. Similarly, metabolites and enzymes involved in glutamine metabolism can be targeted in myeloma cells. Despite preclinical evidence, no clinical trials with metabolism altering agents such as 3BP, DCA, GLUT4 inhibitor (compound 20) and GLS1 inhibitor (compound 968) have been performed as yet with MM patients. However, clinical trials on l-asparaginase combined with Doxil^®^ (PEG-liposomal doxorubicin) and dexamethasone are in phase II trials. Also, clinical trials on 2DG have been completed and it has been shown to have a safe toxicity profile; however, it is not very potent as a single agent, so combination therapy with the proteasome inhibitors could be considered (according to ClinicalTrials.gov).

Promising data have been published involving drug resistance in MM. Resistance to the proteasome inhibitor, bortezomib, has been linked to the upregulation of different metabolic pathways in myeloma cells that can be successfully targeted. Moreover, resistance to the emerging immunotherapeutics has been linked to metabolism. However, resolving this resistance remains an issue since drugs such as 2DG and DCA also have immunosuppressive effects. More investigations on potential other combinations of metabolic drugs and immunotherapeutics would be interesting because of the high specificity and efficacy. Furthermore, immunotherapy can be adapted to each patient allowing for personalized treatment, which would further improve survival. Ideally, agents targeting MM metabolism without affecting the surrounded immune cells, combined with immunotherapy reacting on specific antigens present on MM cells, would be a huge improvement in myeloma treatment. Despite the current developments, MM patients still progress to a certain stage of treatment resistance. Drug resistance can be caused by drug efflux, apoptosis inhibition, drug inactivation, repair of DNA damage due to the drug and epigenetic effects [125]. Another issue is the presence of cancer progenitor cells. These cells are usually drug-resistant and remain present in patients after treatment. Unfortunately, cancer progenitor cells do not only seem to be responsible for cancer relapse; they can also migrate and cause metastasis [125,126,127]. Therefore, the need to investigate drug resistance remains a primary need in MM and other cancers. 

In addition, there are still more metabolic pathways that require investigation in MM, such as the folate pathway and proline metabolism, which shows promising and very interesting targets in its biosynthesis and catabolism pathways. Also, hypoxia related with glucose and glutamine metabolism is an interesting path to explore, together with proline metabolism in MM, to reduce drug resistance and improve survival. An overall understanding of MM cancer metabolism is still needed to improve existing targets and drugs.

## Figures and Tables

**Table 1:** Targets to decrease drug resistance in MM cells. 6-AN, 6-Aminonicotinamide; EGFR, epidermal growth factor; GLS, glutaminase; HKII; hexokinase II; LDHA, lactate dehydrogenase A; PPP, pentose phosphate pathway.

Drug	Target	Resistance Effect in MM	Combination Treatment to Lower Resistance
Bortezomib	Proteasome inhibitor	HKII 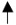	LDHA knockdown
Daunorubicin	DNA-RNA synthesis inhibitor	Glucose metabolism 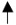	Phloretin
Gefitinib	EGFR inhibitor	PPP 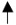	6-AN
Afatinib	EGFR inhibitor	PPP 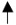	Unknown
CB-839	GLS inhibitor	Glutaminolysis 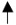	Proteasome inhibitors (bortezomib, carfilzomib, ixazomib, oprozomib)

## Abbreviations

αKG	Alpha Ketoglutarate
ALL	Acute Lymphoblastic Leukaemia
6-AN	6-Aminonicotinamide
ASCT2	Neutral Amino Acid Transporter 2
ATP	Adenosine Triphosphate
BM	Bone Marrow
BMSC	Bone Marrow Stromal Cells
3BP	3-Bromopyruvate
CAM-DR	Cell Adhesion-Mediated Drug Resistance
CAR	Chimeric Antigen Receptor
CHC	α-Cyano-4-Hydroxycinnamic
DC	Dendritic Cell
DCA	Dichloroacetate
2DG	2-Deoxyglucose
DM	Dimethyl
ECM	Extracellular Matrix
EGFR	Epidermal Growth Factor
ERK1/2	Extracellular signal-regulated Kinase
FAD	Flavin Adenine Dinucleotide
^18^F-FDG	Fluorine-labelled Fluorodeoxyglucose
FGF-2	Fibroblast Growth Factor-2
FL	Fas Ligand
GLDH	Glutamate Dehydrogenase
GLS1	Glutaminase 1
GLS2	Glutaminase 2
GPNA	l-γ-Glutamyl-p-Nitroanilide
GS	Glutamine Synthetase
HKII	Hexokinase II
HMCL	Human Myeloma Cell Line
IAP	Inhibitors of Apoptosis Proteins
IGF-1	Insulin-like Growth Factor-1
IL-1β	Interleukin-1β
IL-6	Interleukin-6
IMiDs	Immunomodulatory
JAK	Janus Kinase
LAT1	human L-type Amino acid Transporter 1
LDH	Lactate Dehydrogenase
LDHA	Lactate Dehydrogenase A
MAPK	Mitogen-Activated Protein Kinase
MCT	Monocarboxylate Transporter
MGUS	Monoclonal Gammopathy of Undetermined Significance
MM	Multiple Myeloma
MSO	Methionine Sulfoximine
MYC	Myelocytomatosis oncogene cellular homolog
NAD	Nicotinamide Adenine Dinucleotide
NADH	Nicotinamide Adenine Dinucleotide Hydrogen
NH_4_^+^	Ammonia
NK	Natural Killer
OAF	Osteoclast Activating Factors
OC	Osteoclast
OXPHOS	Oxidative Phosphorylation
PDH	Pyruvate Dehydrogenase
PEP	Phosphoenolpyruvate
PET	Positron Emission Tomography
PI3K	Phosphoinositide-3 Kinase
PK	Pyruvate Kinase
PPP	Pentose Phosphate Pathway
PRPP	5-Phospho-α-Ribosyl-1-Pyrophosphate
RANKL	Receptor Activator of Nuclear factor κB Ligand
SNAT1	Sodium-coupled Neutral Amino acid Transporter 1
STAT3	Signal Transducer and Activator of Transcription 3
TCA	Tricarboxylic Acid cycle
TME	Tumour Micro-Environment
TNF	Tumour Necrosis Factor
TNF-β	Tumour Necrosis Factor-β
TRAIL	TNF-Related Apoptosis-Inducing Ligand
VCAM-1	Vascular Cell Adhesion Molecule 1
VDAC	Voltage-Dependent Anion Channel
VEGF	Vascular Endothelial Growth Factor
